# Supplementary material for: Patient and health system delays in the diagnosis and treatment of tuberculosis in Gandaki, Nepal
Source: PLOS Glob Public Health. 2025 Jun 5;5(6):e0004676. doi: 10.1371/journal.pgph.0004676 (PMC12140256; doi:10.1371/journal.pgph.0004676)
Supplement: S1 File — This file contains the questions used for data collection in this study. (DOCX) [file pgph.0004676.s002.docx]

## Annex VII: Research Questionnaire (English)

**Participant ID____________**

| **TB register review** | | |
| --- | --- | --- |
| TB.1 | Sex | 1. Male  2. Female |
| TB.2 | Age | _____ year  DoB: ______________________ (yy/mm/dd) |
| TB.3 | Smear Results | 1. Positive  2. Negative  3. Not done |
| TB.4 | Nutritional status of the patients | Baseline/initial weight ____________ (Kg)  Height ________ |
| TB.5 | Patient category | 1. PBC  2. PCD  3. EPTB |
| TB.6 | Treatment started | ___________________ date/yy/mm/dd |
| TB.7 | HIV results | 1. Reactive  2. Non-reactive  3. Unknown |

**A. SOCIODEMOGRAPHIC INFORMATION**

| **S.N** | **Questions** | **Answers** |
| --- | --- | --- |
| A.1 | Residence | 1. Urban  2. Rural |
| A.2 | Type of Family | 1. Nuclear  2. Joint/Extended |
| A.3 | Religion | 1. Hinduism  2. Buddhism  3. Islam  4. Christianity |
| A.4 | Ethnicity | 1. Brahmin/Chhetri  2. Janjati  3. Dalit  4. Others |
| A.5 | Marital Status | 1. Currently Married  2.Divorced/Separated  3. Widow/Wider  4. Never Married |
| A.6 | Education | 1. Illiterate  2. Basic level  3. Secondary level  4. Undergraduate level and above |
| A.7 | Occupation | 1. Agriculture  2. Business  3. Service  4. House maker  5. Labor  6. Unemployed  7. Other |
| A.8 | Enrollment in Health Insurance | 1. Yes  2. No |

**B. CLINICAL CHARACTERISTIC AND HEALTH SEEKING BEHAVIOR**

| **S.N** | **Questions** | **Answers** |
| --- | --- | --- |
| B.1 | When was the current illness you are being treated for | 1. ____________/____/____ (yy/mm/dd)  2. I don't know/remember |
| B.2 | What symptoms were appeared before you diagnosed with TB? | 1. Cough  2. Fever  3. Loss of Weight  4. Hemoptysis  5. Chest Pain  6. Night Sweating  7. Breathlessness  8. Loss of appetite  9. Other (specify) ________________________ |
| B.3 | What was/were the first symptom(s) made you seek care? **write codes from Q.B.2**  (More than one response possible and check for that without reading options) |  |
|  | | |
| **B.4** | **When symptoms started, what did you do first ?** | |
| S.N | First action | Mark "X" in front of the patients response |
| 1. | Self-medication |  |
| 2. | Visited drug retail outlets (Pharmacy) |  |
| 3. | Visited traditional healer |  |
| 4. | Visited modern health facility (health post, primary health center, public hospital, private hospital, private clinic) |  |
| 5. | Other (please specify) |  |
|  | | |
| **B.5** | **Formal health facility visited after onset of symptoms?** (ask only when response was modern health facility in Q.B.4) | |
| S.N | Health facility | Mark "X" in front of the patients response |
| 1. | Health Post |  |
| 2. | Primary Health Center |  |
| 3. | Public Hospital |  |
| 4. | Private Hospital |  |
| 5. | Private Clinic |  |
| 6. | Regional Tuberculosis Center |  |
|  | | |
| **B.6** | **Reasons of first consultation of the health facility.** | 1. Accessible  2. Confident getting cured  3. Services available anytime  4. Free services  5. Advised by somebody  6. Referred by previous  7. Others (specify)  _____________________ |
| **B.7** | **Reason for non-consultation with health facility.** (ask only when, response is self-medication, pharmacy and visited traditional healers) | 1. Too far  2. Too busy/long waiting time  3. Bad experience  4. Thought symptoms were not serious  5. Others (specify) __________  ____________________ |
| B.8 | How far is the HCF you visited first from your usual residence? | **____________** minute/hour |
| B.9 | In total how long time, did you spend from the onset of illness to the first HCF visit? | **_______________ days/weeks/months** |
| B.10 | Do you think that you delayed consultation of HCP for your illness? | 1. Yes, if yes go to question B.11  2. No, if no go to B.12 |
| B.11 | If felt delayed, why did you delay consultation of HCP at health facilities? Possible perceived cause of delay | 1. Hoped symptoms would go away by themselves  2. Fear of social isolation  3. Fear of being diagnosed for TB/ Fear of what would be found in diagnosis  4. Fear of routine HIV test at health facilities  5. Lack of money to cover consultation fees  6. Bad staff attitude to patients  7. Perceived poor qualities of health services  8.Busy occupational life intervened in consulting HCF  9. Transport and long distance  10.Tought like common illness 8. Others (mention--------------------------) |
| B.12 | When was your final diagnosis of TB made? | _________________ (yyyy/mm/dd) |
| B.13 | Where was the final diagnosis of TB made? | 1. Health center  2. Public Hospital  3. Regional TB center  4. Private institution  5. Other |
| B.14 | Until the final diagnosis of TB, how many HCF did you visit ? | **______________** (in numbers) |
| B.15 | How many visits did you made to HCF until the final diagnosis of TB? | **______________** (in numbers) |
| B.16 | How long time did it take since you first visit HCF until you were informed you had TB? | **____________________**  (days/weeks/months) |
| B.17 | Do you think that diagnosis of TB was delayed after you made visit to HF? | 1. Failure of providers to reach at diagnosis  2. Lack of facilities/supplies for diagnosis  3. prescription of unnecessary drugs  4. Repeated referral to different facilities  5. Other (specify) ___________ |
| B.18 | Types of diagnostic test used | 1. Microscope  2. Chest X-ray  3. Microscope and Chest X-ray  4. Gene Xpert  5. Others |
| B.19 | Do you have history of TB contact in the last one year? | 1. Yes (if yes, go to B.20)  2. No |
| B.20 | Point of contact | 1. Households  2. In the facility  3. At work  4. School/College  5.Others |

**C. ANTI-TB TREATMENT PRACTICE**

| **S.N** | **Question** | **Options/response** |
| --- | --- | --- |
| C.1 | When did you start anti-TB treatment? | 1. _____________ yy/mm/dd  2. I don't know/remember |
| C.2 | How long it takes you to start TB treatment since first HCP consultation. | ___________________  days/weeks/months |
| C.3 | How long after the diagnosis of TB that you commenced anti-TB treatment? | 1. Immediately  2. _______________  days/weeks (go to C.4) |
| C.4 | Why you did not start treatment immediately? | 1. I was reluctant to initiate the treatment  2. Fear of long treatment  3. Lack of anti-TB drugs at the facility  4. Absence of DOTS provider  5. Failure to present treatment supporter  6. Inability to arrange accommodation at nearby  7. Too ill to initiate early  8. Other (specify-- |

**D. KNOWLEDGE AND PERCEIVED STIGMA RELATED TO TUBERCULOSIS**

| **S.N** | **Questions** | **Options/Response** |
| --- | --- | --- |
| D.1 | Have you previously heard about Tuberculosis (TB)? | 1. Yes  2. No |
| D.2 | Source of information on TB | 1. Media  2. Health facility/Health care providers  3. Education  4. Friend/relatives  5. Others |
| D.3 | Correctness of information on TB  **(use these code: Yes/right= 0, No/wrong=1 and Don’t know=2)** | |
| **S.N** | **Questions** | **Response** |
| D.3a | What kind of disease do you have? |  |
| D.3b | Is TB hereditary? |  |
| D.3c | Is TB contagious? |  |
| D.3d | Is TB curable? |  |
| D.3e | Dou you know if there is a vaccine for TB? |  |
| D.3f | Do you know the approximate duration of Treatment? |  |
| D.3g | Do you know the kind of TB drugs? |  |
|  | | |
| **D.4** | **TB Stigma**  **(Strongly agree= 0, Agree= 1, Average= 2, Do not agree= 3, Do not agree at all= 4 )** | |
| D.4a | Do you feel ashamed for having TB? |  |
| D.4b | Do you have to hide your TB diagnosis from the other people? |  |
| D.4c | Does TB affect relationships with others? |  |
| D.4d | Is TB very costly due to the long duration of the disease? |  |
| D.4e | Do you prefer to live isolated since you got TB diagnosis? |  |
| D.4f | Does the TB affect your work performance? |  |
| D.4g | Does TB affect marital relations? |  |
| D.4h | Does TB affect family responsibilities? |  |
| D.4i | Do you think there are fewer chances of marriage due to a TB diagnosis? |  |
| D.4j | Does TB affect your family relations? |  |
| D.4k | Does TB cause female infertility? |  |
| D.4l | Does TB lead to serious complications during pregnancy? |  |
| D.4m | Does TB affect breastfeeding? |  |
| D.4n | Does TB affect pregnancy outcomes? |  |
| D.4o | Is a girl unable to decide for getting TB treatment? |  |

**E. BEHAVIORAL CHARACTERISTICS**

| **S.N** | **Questions** | **Answers** |
| --- | --- | --- |
| E.1 | Smoking status | 1. Never  2. Current smoker  3. Quitted smoker |
| E.2 | Alcohol use status | 1. Never used  2. Past user  3. Current user |
| E.3 | Co-existence of chronic diseases  (Other than HIV/AIDS) | 1. Yes  2. No |

**Thank for your valuable time!!!**
