# Supplementary material for: Patient and health system delays in the diagnosis and treatment of tuberculosis in Gandaki, Nepal
Source: PLOS Glob Public Health. 2025 Jun 5;5(6):e0004676. doi: 10.1371/journal.pgph.0004676 (PMC12140256; doi:10.1371/journal.pgph.0004676)
Supplement: Attachment — Submitted filename: Response to the editor and reviewers.docx [file pgph.0004676.s005.docx]

**Journal requirements:**

1. Please provide a complete Data Availability Statement in the submission form, ensuring you include all necessary access information or a reason for why you are unable to make your data freely accessible. If your research concerns only data provided within your submission, please write "All data are in the manuscript and/or supporting information files" as your Data Availability Statement.

**Authors Response:** All relevant data are within the manuscript and its supporting information files.

2. Please review your reference list to ensure that it is complete and correct. If you have cited papers that have been retracted, please include the rationale for doing so in the manuscript text, or remove these references and replace them with relevant current references.

**Authors Response:** we have thoroughly reviewed our reference list and confirm that no retracted papers were cited. All references are complete and accurate.

**Responses to reviewers**

**Manuscript ID:** PGPH-D-24-01716

**Title:** Delays in Diagnosis and Treatment among Tuberculosis Patients in Gandaki, Neal

Dear Dr. Prabin Karki,

Thank you for your thorough review and valuable feedbacks on our manuscript. We addressed all the comments and made the following revisions:

| **Reviewers comment** | **Authors Response** |
| --- | --- |
| Title: This study title and objectives are to identify factors that delays in diagnosis and treatment but your results incorporate patients delay and health system delay. You have a figure 1 that shows diagnosis delays and treatment delays as others delay as well. Also, you have total delays which includes all of it. How do you correlate? It’s a bit confusing. Please provide detail justification. | Thank you for your feedback. As shown in figure 1, both patient and health system delays are the part of delay in diagnosis and treatment. We defined these delays as, 1. Patient Delay: The time interval between first symptom onset to first health seeking behavior or HCF consultation.  2. Health System Delay: The time interval between first HCF consultation to date of treatment initiation.  3. Total Delay: This is the sum of Patient Delay and Health System Delay.  To address your concerns and improve the clarity, we have revised our manuscript title and objective to  ***"Patient and Health System Delays in the Diagnosis and Treatment of Tuberculosis in Gandaki, Nepal."*** *(Line 1-2)*  **Objectives:** *To assess patient and health system delays in diagnosis and treatment of TB in Gandaki Nepal. (Line 12-13)* |
| Line 123: Only DOTS can be used after illustrating its full form in previous lines. | Thank you, we have defined DOTS at its first occurrence *(Line 120)* and used consistently thereafter *(Line 126, 127).* |
| Line 126: Why did this study excluded the participants whose treatment failed? Justify. | Thank you for your question. This study excluded TB patients whose treatment failed for several reasons. These patients might have other health issues or reasons for their treatment failure, such as drug resistance, co-morbidities, or previous incomplete treatment. Including them could distort the common reasons for delays that most patients face.  Additionally, patients with failed treatment status require retreatment, making it difficult for them to accurately remember key dates such as symptom appearance, treatment sought, diagnosis, and treatment initiation. This could introduce errors and biases in the data. By excluding these patients, the study aims to provide a clearer understanding of the typical patient experience and more reliable data. |
| Line 130: what was the type of delay from previous study used? Was it delay in seeking care or receiving treatment? | The proportion used for sampling was Patient delay in previous study (Delay in Seeking Care) |
| Line 134: Provide the number of participants from 10 DOTS centers in supplementary file. | Comment accepted, and we have now provided number of participants from 10 DOTS centers in supplementary file. |
| Line 153: You have used extensive literature review. Was it that extensive? You can remove the word extensive. | Comment accepted. We have now removed the term extensive from both abstract *(Line 17)* and methodology section *(Line 157)* of manuscript. |
| Line 155: Were the tools used was validated in Nepali language? If yes cite it. If not, how do you maintain the validity of the tools in Nepali language? | No. tools used in this study was not pre-validated in Nepali language. However, to maintain the validity of the tools in Nepali language we took several steps which include back translation (English-Nepali-English) of questionnaire, pre-testing of tools, brief orientation on tools and data collection procedures to data collectors and expert consultation. |
| Line 179: How did your data collectors (public health students) maintain the credibility of responses for clinical characteristics? How was it done? Justify. | Thank you for raising interest in this matter. Initially data collectors were oriented on study tools, and how to collect data. The Clinical characteristics of the patients were type of TB present (pulmonary vs extra pulmonary), presence of HIV comorbidity, presence of other chronic disease comorbidities, type of health facility at which the final diagnosis was made, history of contact with TB-infected people in the last year and place of contact and smear status (positive and negative). This information was collected from Health Information Management System (HMIS) 6.4 A (TB treatment card- Health facility) and HMIS 6.4 B (TB treatment card- Patient).  We have added statement regarding this in our manuscript.  (*Line 164-165) The data collectors were oriented on study tools and data collection procedures before initiating field work.* |
| Line 187: Cite the reference. Was this scoring validated from standard tools? | Relevant reference was cited. Yes, it was validated scoring method |
| Line 192: Again, cite the relevant reference for the Likert scale used. | Relevant reference was cited. |
| Line 247: Is it P value less than or equals 0.05? Please check. If yes, please explain? | Thank you for your concern regarding p-value. yes, it is p<0.05. In our study, included variables with a p-value <0.05 from the binary logistic regression analysis for inclusion in the multivariable logistic regression model. As this method is a well-established and widely accepted statistical practice. By setting this threshold, we ensured the variable included in the multivariable logistic regression model are those that have a significant association with the outcome in the binary logistic regression. |
| Line 249: Report the highest VIF value from your data. | Thank you, the highest VIF value was reported in Manuscript. The statement now looks like, *Multi collinearity among the independent variables was assessed using variance inflation factor (VIF) analysis. The VIFs for the independent variables were less than two, with the highest VIF observed was 1.88 for the variable "education status". (Line 254-256)* |
| Line 302: The table is large. Split the table and use different table headings. If some characteristics are multiple response, use notation for it. | Thank you, we have splited table and used different table heading for splited tables.  Table 2 clinical characteristics and health seeking behavior among tuberculosis patients in Kaski, district (n=194) is splited into Table 2 and Table 3.  Table 2 Clinical behavior characteristics tuberculosis patients in the Kaski district (n=194) - Line 304  Table 3 Health seeking behavior among Tuberculosis patients in Kaski, District (n=194) - Line 306  Multiple responses were given notation for it as needed. |
| Line 308: Use only DOTS. | Thank you, Comment accepted. We used DOTS |
| Line 310: Table 3. Note as multiple response where necessary. Check all tables again. | Comment accepted, and noted multiple response where necessary in all tables. |
| Line 323: Figures are missing. Check it. | Thank you for information we have included the missing figure in manuscript.  *Line 177-179, Delays in Diagnosis and Treatment among Tuberculosis patients*  *Line 127-128, Delays in Diagnosis and Treatment among Tuberculosis Patients in Kaski, District* |
| Line 282-385: Cite proper references. | Thank you for your feedback. Now we have cited relevant references in our manuscript. We have included references for tools adopted, scales used for knowledge level and perceived stigma level *(Line 508)*. In addition to this citation [38, 39, 40 and 41] are added in to discuss about private sector engagement *(Line 582-592)*.  Reference [48, and 49] is also added to discussion about non-enrollment in health insurance program. |

Dear Professor Dr. Sachin Atre,

We sincerely appreciate your valuable time in reviewing this manuscript. We Thank you for your feedback and comments. After careful review, we have now addressed all of your comments and accepted feedbacks. Kindly find comment wise response in table below.

| **Reviewers Comment** | **Authors Response** |
| --- | --- |
| 1. Abstract: The first sentence in the background should not directly link to increased drug resistance. It may lead to that in some cases such as where patients undergo incomplete treatments. Mere delays will not lead to increased resistance. It needs to be worded carefully. | Comment accepted and revised the background. **Abstract**  **Background:** *Delays in accessing healthcare services worsen disease, leading to treatment failure, increased mortality, and higher community transmission rate of tuberculosis (TB). While prolonged delays may contribute to drug-resistant TB strains in some cases, this study aimed to assess the delays in TB diagnosis and treatment and the associated factors among TB patients in the Gandaki, Nepal.* |
| 2. Line 15: multi-country | Thank you for correction. Comment accepted and we revised the term in manuscript. |
| 3. Patient and health system delays need to be calculated separately. Even after reaching the health system (first point as private), further delays in diagnosis/treatment may be due to patients’ behaviour. It seems there is an overlap. Patient related delays are more likely linked to socio-economic status, stigma, illiteracy etc. as seen from the results, whereas health system delays are more likely related to system related issues. They both need different kinds of interventions. The multivariate analysis looks more appropriate. | Thank you for your concern regarding health system delay, we agree with you and we also believe diagnosis/treatment delay could be due to patient behavior as well. That's why we have already mentioned this in limitation section of our manuscript. *(Line 481-483) The current study explored health system-related factors only in TB patients. Thus, further studies can be conducted to explore factors contributing to patient delay and health system delay from service providers and system perspectives.* |
| 4. Conclusion: Needs to be more specific than just education/training. Factors that caused significant delays, should be focused while suggesting the intervention measures. Private providers’ lack of suspicion of TB is one of the reasons for delayed diagnosis and treatment. | Thank you for feedback, we have now revised recommendations in conclusion as you suggested.  *Line 37-41*  ***Conclusion:*** *Patient and health system delays were prevalent among TB patients. Reducing these delays is crucial for improving TB control. Therefore, urgent action is needed to implement education campaigns to improve TB literacy. Additionally, engaging private and informal healthcare providers and enhancing their capacity to deliver timely and effective TB care could potentially mitigate delays in diagnosis and treatment.* |
| 5. Line 89 should be GeneXpert or Xpert assay. | The comment is accepted and we have now made it GeneXpert in *Line* 93 |
| 6. Lines 98-99: The rationale is unclear. Can authors make it explicit? | Thank you for the suggestion, now the rationale was revised. |
| 7. Lines 108-112: Reference needs to be provided. Authors can provide the reference in the first sentence and mention about the same report---etc. | The comment is accepted and done as suggested. |
| 8. Line 273: 35.6% cannot be the most. Better to say around a third | The comment is accepted. We revised the sentence as suggested. Now the revised sentence looks like,  *Line 279, Education attainment varied; around a third (35.6%) had completed Grade 9-12, while almost two-thirds (32.47%) had never received formal education.* |
| 9. Lines 295-298: It is commonly observed in S. Asia that majority patients approach private health facility and over two third patients reported visits to >3 providers before getting TB diagnosis. The same experience was reported in India (Re. Atre et al. 2022 AJRCCM paper on pathways to TB and MDR-TB care). | Thank you for your suggestion, we have now added a short paragraph of this in our manuscript and added new references. The added paragraph is mentioned below.  *Line (375-380), In our study it was observed that patients who sought care from non-NTP providers were three times more likely to experience delays compared to those who consulted NTP providers. Despite this more than 45% patient sought care from private health care providers, similar experience was documented in studies conducted in India[38, 39]. Most of patients in South Asia reflect similar health seeking behavior as private sector providers are more accessible and available[40, 41], and ensure confidentiality that offers protection against stigma[39].* |
| 10. Can authors elaborate reasons for patients’ non-enrolment in Govt insurance program? | Thank you for your suggestion. We have now included a brief paragraph to address this comment in our manuscript.  *(Line 412-417) Several factors contributed to non-enrollment in governments health* *insurance program, including lower household income, absence of chronic illness within the household, inappropriate benefit packages, cultural beliefs, and affordability issues[48, 49]. The consistency in these findings highlights that socio-economic and cultural factors may play significant influence health insurance enrollment decisions across different geographic and demographic contexts.* |
